# A new path to spillover: MHC-II entry of influenza A viruses

**DOI:** 10.1371/journal.pbio.3003550

**Published:** 2025-12-08

**Authors:** Silke Stertz, Umut Karakus

**Affiliations:** Institute of Medical Virology, University of Zurich, Zurich, Switzerland

## Abstract

Spillover of influenza A viruses from animals to humans represents a threat to our health. This Perspective discusses emerging research that suggests some influenza A viruses can enter host cells via MHC-II across species, reflecting on the consequences that this may have for virus spillover risk.

## Teaser

Zoonotic spillover of influenza A viruses represents a threat to human health. Emerging research suggests that some influenza A viruses can enter host cells via MHC-II across species, potentially increasing spillover risk.

Respiratory virus infections place a significant burden on human health. Influenza A (IAV) and B viruses alone account for approximately 1 billion infections annually, leading to 3–5 million severe cases requiring hospitalization [1]. Furthermore, the threat of a respiratory virus pandemic remains ever-present. Containing outbreaks is particularly challenging due to the limited understanding of airborne transmission and the scarcity of effective countermeasures [2]. While attention had shifted to coronaviruses after the COVID-19 pandemic, focus is returning to IAV due to the expanding host range and global spread of highly pathogenic avian H5N1 strains. Particularly concerning is the recent emergence of H5N1 in dairy cattle in the USA, which has raised fears of a potential H5N1 pandemic [3].

So how can we mitigate such zoonotic threats? First, we need to identify and assess them before devising mitigation plans. This requires studying past zoonotic events to deduce the mechanisms of adaptation. Different viral features, such as viral polymerase activity or acid stability of the viral surface glycoprotein hemagglutinin, contribute to zoonotic potential. Here, we focus on receptor specificity, crucial for zoonotic spillover, as one of the key determinants [4]. Most IAVs use sialic acid, the terminal sugar on many host cell glycans, as their primary attachment receptor, mediated by viral hemagglutinin. Mammalian IAVs prefer sialic acid linked to galactose via an α2,6′-linkage, whereas avian IAVs bind sialic acid with an α2,3′-linkage. Successful adaptation from avian to mammalian hosts therefore requires a switch in sialic acid specificity.

But what if IAVs could use a different receptor? Our previous work revealed that the IAV subtypes H17N10 and H18N11, which have so far been detected exclusively in bats, use major histocompatibility complex class II (MHC-II) molecules as entry receptors instead of the conventional sialic acid receptors [5]. As a central immune regulator, MHC-II is abundantly expressed on professional antigen-presenting cells and in certain subsets of airway epithelial cells, making it a good target for respiratory viruses. Interestingly, this ability to exploit MHC-II is not limited to bat IAVs: the newly identified avian H19 subtype also lacks the ability to bind sialic acid and instead uses MHC-II [6]. Intriguingly, both avian and human strains of the IAV H2 subtype exhibit dual receptor usage; they can enter cells either via MHC-II in a sialic acid-independent manner or through the traditional sialic acid pathway [7]. Perhaps most striking is the breadth and specificity of MHC-II usage across host species. H17, H18, H19, and H2 IAVs can use MHC-II complexes found in various aquatic birds, such as ducks and swans. However, only H17, H18, and H2 (but not H19) are capable of entering cells via human and swine MHC-II. The molecular determinants of this difference in breadth are not understood as the binding sites are only partially defined for a small subset of HAs and HLA-DR [7,8].

**Fig 1:**
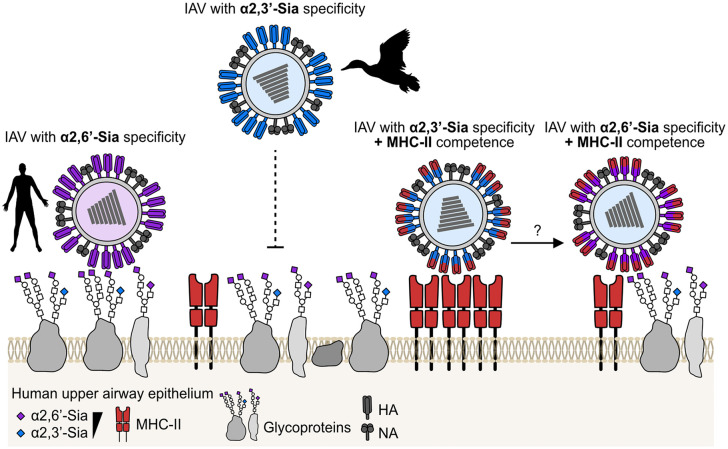
MHC-II entry competence of IAV as a novel path to zoonotic spillover. Human influenza A viruses (IAVs; purple) have adapted to use α2,6′-linked sialic acid (Sia) as host cell receptors, which are highly abundant in the human upper respiratory tract. By contrast, avian IAVs (blue), which preferentially bind to α2,3′-linked Sia, face a major barrier to spillover because of the limited availability of these receptors in the human upper airways. This restriction could potentially be overcome if avian IAVs possess the ability to bind human major histocompatibility complex class II (MHC-II) molecules, which are expressed on cells of the respiratory epithelium. Thus, MHC-II competence (highlighted in red) may enhance the zoonotic potential of avian IAVs by facilitating their initial spillover into humans and promoting subsequent adaptation to human-type α2,6′-linked Sia receptors. The icons for humans and ducks were obtained from https://www.phylopic.org.

Given the established role of receptor specificity in zoonotic transmission, and the fact that avian H2 IAVs can use human MHC-II for entry, these findings raise the possibility that MHC-II tropism represents a previously unrecognized determinant of zoonotic risk. Support for this hypothesis comes from an earlier study that assessed the replicative potential of a broad panel of avian H2 viruses in mammalian systems. The authors of this study observed large differences in infectivity and replication that could not be explained by known zoonotic markers [9]. However, when we compared their data with our mapped sequence motif for MHC-II entry competence in H2 viruses, a clear positive correlation emerged [7]. Thus, the ability to use MHC-II as receptor does indeed seem to promote IAV’s ability to infect and replicate in mammalian cells and should be considered for zoonotic risk assessment (Fig 1).

In light of this, it is therefore conceivable that MHC-II entry competence contributed to the zoonotic transmission of H2 IAV, which led to the 1957 H2 pandemic. But what about H1 and H3 IAVs, the two other subtypes that successfully made the jump to humans? So far, MHC-II entry competence has not been observed in the tested panel of human and avian strains of H1 and H3 IAVs, suggesting that MHC-II competence is not widespread among these subtypes. However, fewer than 10 strains of each subtype have so far been tested, so it is still possible that certain H1 or H3 strains could exhibit MHC-II entry competence. Furthermore, testing has primarily focused on human MHC-II, and it is possible that some strains can utilize MHC-II molecules from a limited subset of species, as seen with H19 strains. Even such restricted MHC-II usage could influence host tropism and facilitate adaptation in intermediate hosts. In support of this idea, a recent preprint identified MHC-II entry competence in a swine H3 strain [10]. It will therefore be crucial to test H1 and H3 viruses more comprehensively against a broad range of MHC-II complexes, particularly those strains that are closely related to pandemic viruses, to determine if MHC-II entry competence plays a more general role in zoonotic transmission. Current and future zoonotic threats, such as the widely circulating H5N1 IAVs of clade 2.3.4.4b, should also be tested for MHC-II entry competence. Given the broad host range of these viruses, it will be worth investigating whether MHC-II entry competence contributes to their success in crossing species barriers.

Beyond the prevalence and species specificity of MHC-II entry competence, questions also arise about the impact of dual receptor specificity within the host. MHC-II usage could influence IAV tropism and its interplay with the host immune system, as susceptibility to H2N2 infection is enhanced in MHC-II-expressing immune cells and airway epithelial cells [7]. Another key question that remains unanswered is whether MHC-II usage could impact the host immune response or even facilitate immune evasion, for example by modulating receptor availability or impairing antigen-presenting cell functions. Unlike conventional IAVs that rely on neuraminidase to cleave sialic acid receptors and promote viral release, an alternative receptor-destroying activity targeting MHC-II would represent a distinct adaptation of such viruses. Addressing these questions could reveal new intersections between receptor usage, immune regulation, and viral evolution, reshaping our understanding of how influenza viruses adapt and emerge across hosts.

Together, these open questions highlight that MHC-II entry competence may represent an underappreciated feature of IAV biology with implications for host range and zoonotic potential. A systematic study of MHC-II usage across subtypes, host species, and cellular contexts combined with evolutionary and immunological approaches will be needed to reveal how MHC-II entry competence contributes to cross-species transmission and to identify viral vulnerabilities that might be exploited for surveillance and intervention.

## Abbreviations

**:**
IAV: influenza A virus
MHC-II: major histocompatibility complex class II
